# A return-on-investment approach for prioritization of rigorous taxonomic research needed to inform responses to the biodiversity crisis

**DOI:** 10.1371/journal.pbio.3001210

**Published:** 2021-06-01

**Authors:** Jane Melville, David G. Chapple, J. Scott Keogh, Joanna Sumner, Andrew Amey, Phil Bowles, Ian G. Brennan, Patrick Couper, Stephen C. Donnellan, Paul Doughty, Danielle L. Edwards, Ryan J. Ellis, Damien Esquerré, Jéssica Fenker, Michael G. Gardner, Arthur Georges, Margaret L. Haines, Conrad J. Hoskin, Mark Hutchinson, Craig Moritz, James Nankivell, Paul Oliver, Carlos J. Pavón-Vázquez, Mitzy Pepper, Daniel L. Rabosky, Kate Sanders, Glenn Shea, Sonal Singhal, Jessica Worthington Wilmer, Reid Tingley

**Affiliations:** 1 Department of Sciences, Museums Victoria, Melbourne, Australia; 2 Department of Biology, Washington University, St. Louis, MI, United States of America; 3 School of Biological Sciences, Monash University, Clayton, Australia; 4 Division of Ecology & Evolution, Research School of Biology, Australian National University, Canberra, Australia; 5 Biodiversity & Geosciences Program, Queensland Museum, Brisbane, Australia; 6 Snake & Lizard Red List Authority, CI-IUCN Biodiversity Assessment Unit, IUCN North America Office, Washington, DC, United States of America; 7 South Australian Museum, North Terrace, Adelaide, Australia; 8 Collections & Research, Western Australian Museum, Welshpool, Australia; 9 Department of Life & Environmental Sciences, University of California, Merced, Merced, CA, United States of America; 10 Biologic Environmental Survey, East Perth, Australia; 11 College of Science & Engineering, Flinders University, Adelaide, Australia; 12 Institute for Applied Ecology, University of Canberra, Canberra, Australia; 13 College of Science & Engineering, James Cook University, Townsville, Australia; 14 School of Biological Sciences, University of Adelaide, Adelaide, Australia; 15 Environmental Futures Research Institute, Griffith University, Australia; 16 Museum of Zoology & Department of Ecology and Evolutionary Biology, University of Michigan, Ann Arbor, MI, United States of America; 17 School of Veterinary Science, University of Sydney, Sydney, Australia; 18 Australian Museum Research Institute, The Australian Museum, Sydney, Australia; 19 Department of Biology, California State University, Dominguez Hills, Carson, CA, United States of America; Princeton University, UNITED STATES

## Abstract

Global biodiversity loss is a profound consequence of human activity. Disturbingly, biodiversity loss is greater than realized because of the unknown number of undocumented species. Conservation fundamentally relies on taxonomic recognition of species, but only a fraction of biodiversity is described. Here, we provide a new quantitative approach for prioritizing rigorous taxonomic research for conservation. We implement this approach in a highly diverse vertebrate group—Australian lizards and snakes. Of 870 species assessed, we identified 282 (32.4%) with taxonomic uncertainty, of which 17.6% likely comprise undescribed species of conservation concern. We identify 24 species in need of immediate taxonomic attention to facilitate conservation. Using a broadly applicable return-on-investment framework, we demonstrate the importance of prioritizing the fundamental work of identifying species before they are lost.

## Abstract 2

In order to inform conservation effort, there is urgent need for rigorous taxonomic research to describe species under threat of extinction. Implementation of a new prioritization method identified 282 Australian reptile species needing taxonomic research, of which 17.6% represent undescribed species of conservation concern; this approach could be readily implemented across many faunal groups.

## Introduction

Species-level listings of fauna and flora are the foundation of conservation management globally (e.g., the IUCN Red List). While most agencies have some provision for conservation units below the species level, ambiguity around definitions and lack of formal recognition of management units means these alternate taxonomies are not commonly employed or enforced [1]. Yet close inspection of many, if not most, highly diverse fauna and flora groups shows that significant work is needed to provide rigorous and comprehensive taxonomic documentation.

This problem is most profound in megadiverse groups, with recent estimates suggesting less than 20% of terrestrial arthropods have been described [2,3]. However, even in vertebrates, the extent of taxonomic uncertainty is astounding. New species are still being discovered, but undocumented diversity hidden within described “species” still looms large [4]. This hidden diversity is invisible to conservation assessment. These undescribed species often have small geographical ranges [5,6], making them of particular conservation concern because distribution size is a strong predictor of extinction risk [7].

Globally, there are many examples across all faunal groups of the impact of incomplete taxonomy in the conservation of species [8–15], with clear evidence that taxonomic research improves conservation efforts and outcomes [8,16]. Here, we provide an objective, transparent, and data-driven approach to ascertain which species are in greatest need of rigorous taxonomic research for subsequent conservation management. Using a return-on-investment approach [17], a well-established methodology used in conservation biology but not yet in taxonomy, we establish a framework to assess taxonomic need and prioritize species for more rigorous taxonomic research. We implement this method in Australian squamate reptiles, an ideal test case both because it is a highly diverse fauna and because it has been studied extensively by many taxonomic experts. However, our approach is readily transferrable to most organismal groups.

Squamate reptiles (lizards, snakes, and amphisbaenians) are the most diverse order of terrestrial vertebrates, with more than 10,000 currently recognized species. Of the global squamate diversity, Australia stands out with more than 1,000 species (approximately 10% of global diversity). Moreover, Australia is a lizard-diversity hotspot [18] with very high endemicity: 98% of squamates species are found only in Australia [19]. The number of recognized Australian squamates has increased by 40% in the last 40 years [19]; a much larger increase than in any other Australian terrestrial vertebrate group. However, expert accounts suggest there are still significant levels of undescribed diversity in Australian squamates, with important implications for conservation.

We brought together experts in the taxonomy and systematics of Australian squamates, spanning researchers using both genetic and morphological approaches, to undertake structured expert elicitation to quantify outstanding taxonomic uncertainties in Australian squamates—the “known unknowns.” We compiled a dataset identifying species for which further taxonomic research is required, what research effort is required to complete species descriptions (the taxonomic process of species recognition), and the predicted conservation concern for the resulting new taxa (referred to as “candidate” species). We implemented a deliberately conservative approach for estimating the number and conservation concern of candidate species, ensuring a high level of confidence that prioritized taxa are of immediate need of rigorous taxonomic research.

## Results and discussion

### Expert assessment of taxonomic need

Of 1,034 Australian squamate species, we were able to assess the taxonomic status of 870 (84.1%), with 282 of these probably or definitely needing taxonomic revision (S1 Table). The majority of these taxonomic revisions (216 of 282) would lead to an increase in species number, while another 43 species belong to large complexes where boundaries across current and candidate species are too complicated for clear-cut assessment of net increase or decrease in diversity. The remaining 11 species would lead to synonymy (amalgamation of 2 species into one). These results indicate that 24.8% (29.8% if large species complexes are included) of Australian squamate reptiles comprise additional, unrecognized species.

This potential increase in diversity was high across most squamate groups, and highest in 4 Australian squamate families (Diplodactylidae, Varanidae, Pythonidae, and Typhlopidae) that were each identified as having ≥30% of species requiring taxonomic revision. Although not as diverse as the squamates, an assessment of the remaining Australian reptiles (turtles and crocodiles) found a similar level of taxonomic uncertainty in freshwater turtles, with 26.1% (6 species) needing taxonomic revision that would lead to an increase in diversity (S2 Table).

Our results suggest that the current IUCN estimate of 6.3% (Methods) of Australian squamates requiring taxonomic revision is a significant underestimate. While the IUCN assessment process recognizes the importance of taxonomic uncertainty to accurately assess the extinction risk of a species, its primary focus is on assessing species as currently recognized. Given most diverse faunal groups likely harbor similar, if not greater, levels of taxonomic uncertainty [20], this approach clearly underestimates the scale of taxonomic issues.

To further explore the conservation implications of the unrecognized taxonomic diversity of Australian squamates, we scored three conservation elements for each species group (Methods): highly localized species (used as a proxy for vulnerability to threatening processes [9]); probable threatening processes (i.e., factors that may cause candidate species to become threatened); and a high probability of an IUCN Red List threatened status (i.e., already within a threatened species group). There were 52 species (24.0%) needing taxonomic revision that were identified as containing unrecognized highly localized species, while 38 (17.6%) were conservatively identified as harboring undescribed species that would be of conservation concern.

Our estimate that 17.6% of undescribed species may be of conservation concern is close to the global average (approximately 18%) of threatened squamates [19]. Currently, 7.1% (range 6.8% to 11.3%, depending on the treatment of Data Deficient species) of Australian squamates are of conservation concern [19]. It has been postulated that the low estimate of threatened squamates in Australia may be due to limited knowledge of population sizes, trends, and threats to which they are exposed, rather than a lower degree of imperilment [19]. Our study suggests that the low level of currently recognized threatened squamates in Australia may also be attributable to unresolved taxonomic issues leading to imperiled species being invisible to conservation assessment. Indeed, 4 out of the top 5 most imperiled reptile species in Australia were only described in the last decade [21].

**Fig 1:**
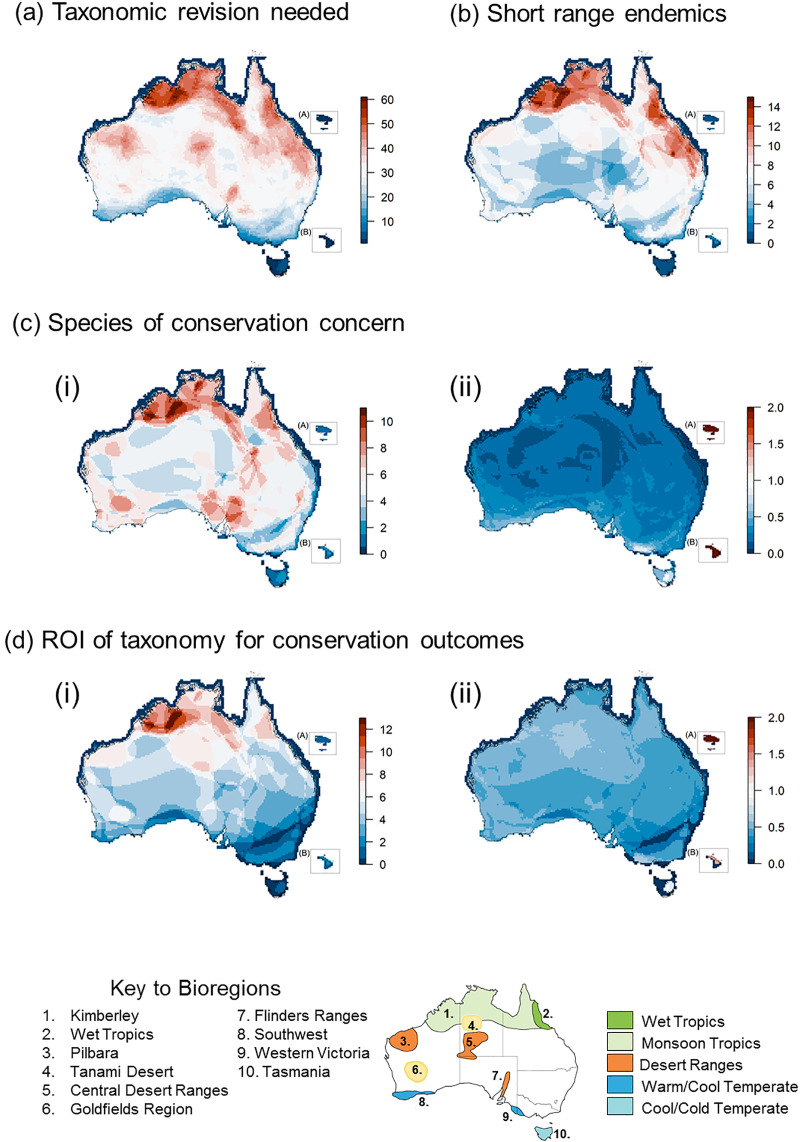
Spatial pattern of species richness in Australian lizards and snakes. **(a)** The distribution of species requiring taxonomic revision that would result in an increase in species. **(b)** Species requiring taxonomic revision that contain undescribed highly localized species. **(c)** Species requiring taxonomic revision that contain species of conservation concern. **(d)** Priority species (*N =* 24 identified using a return-on-investment (ROI) analysis. Maps **(a)** and **(b)** present the sum of species contained in each 25 × 25 km cell. For the last 2 criteria—**(c)** and **(d)**—data are mapped in 2 ways: (i) sum of species in cells and (ii) weighted mean. The weighted mean ROI for map cells are square root transformed, due to significant influence of extreme values. Scale for each map indicates range of cell scores. Insets (not to same scale) show Norfolk Island group (A) and Lord Howe Island group (B). A key to bioregions is provided below maps, with regions referred to in text numbered. Assessment data presented in maps available in S1 Data and distributional data used for mapping was collated by Tingley and colleagues [19] and is publicly available from the IUCN website (https://www.iucnredlist.org/). Map layer: Bioregional Assessment Source Dataset (https://data.gov.au/data/dataset/0cb242e2-daed-4507-a42e-73892c0941a1).

We also visualized the spatial patterns of candidate species richness by overlaying species geographic range maps on a 25 × 25 km grid of Australia (Methods). The distribution of all species identified as requiring taxonomic revision reflects the richness of currently recognized species [19], with greatest richness in the northern Australian Monsoon Tropics, Wet Tropics, Central Desert Ranges, and the Pilbara (Fig 1A, including key for bioregions). While these regions already host the highest species richness, with approximately 120 species in some grid cells [19], some areas comprise more than 40 species (>50 species in the Kimberley region in north-western Australia) that need further taxonomic research.

The geographic distribution of highly localized candidate species shows high species richness in the Australian Monsoon Tropics, particularly the Kimberley region, Wet Tropics, and mid-east Queensland (Fig 1B). In contrast, the geographic distribution of candidate species of probable conservation concern shows the highest mean values in southern Australia, particularly in Tasmania, western Victoria, Lord Howe Island, and Norfolk Island, even though there are more candidate species of conservation concern in the Australian Monsoon Tropics, Wet Tropics, Flinders Ranges, and Goldfields region (Fig 1C). This result largely reflects the current distribution of threatened species [19], where islands and south-eastern regions have lower species diversity but a heightened level of imperilment.

### Return-on-investment analyses to prioritize taxonomic research

**Fig 2:**
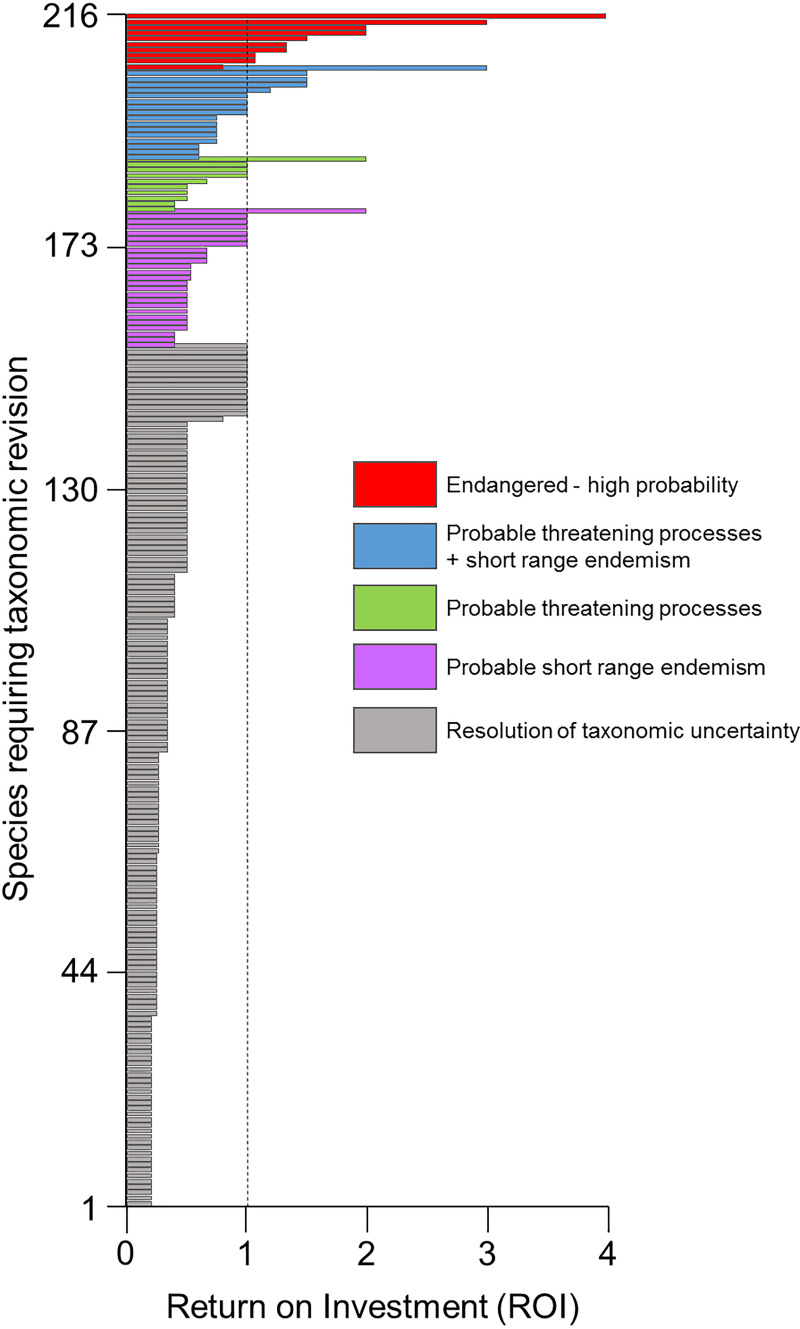
Estimated return on investment (ROI) for taxonomic research focused on conservation outcomes of Australian lizards and snakes. Each horizontal bar represents a species complex that experts assessed as needing taxonomic revision and that would likely lead to increased diversity. Species are colored according to the predicted conservation-related outcome of taxonomic revision. ROI is a cost-effectiveness metric (Methods), which estimates conservation concern of a species in relation to the research required to complete taxonomic revision. An ROI >1 (depicted by the dashed line) indicates high conservation concern in comparison to research needed. Assessment data underlying figure available in S1 Data.

We then used the data to prioritize taxonomic revisions for enhanced conservation outcomes, employing a return-on-investment (ROI) analysis. We categorized the research resources (e.g., human effort, financial expense) needed to complete rigorous taxonomic revisions for species in relation to the level of likelihood a given species contains undescribed taxa that would be of conservation concern (Methods). A resulting ROI metric >1 indicates a species that has high predicted conservation importance but lower research investment needed to complete rigorous taxonomic revision. Our analysis identified 24 species of lizards with an ROI ≥1 (Fig 2). The highest number of these species occur in the Kimberley region of the Australian Monsoon Tropics (Fig 1D); however, regions with high mean ROI values also include the offshore islands (Tasmania, Lord Howe and Norfolk Island groups), western Victoria, and the Tanami Desert region in northern Australia (Fig 1D). We suggest that all these regions are an immediate priority for conservation-focused taxonomic research.

**Fig 3:**
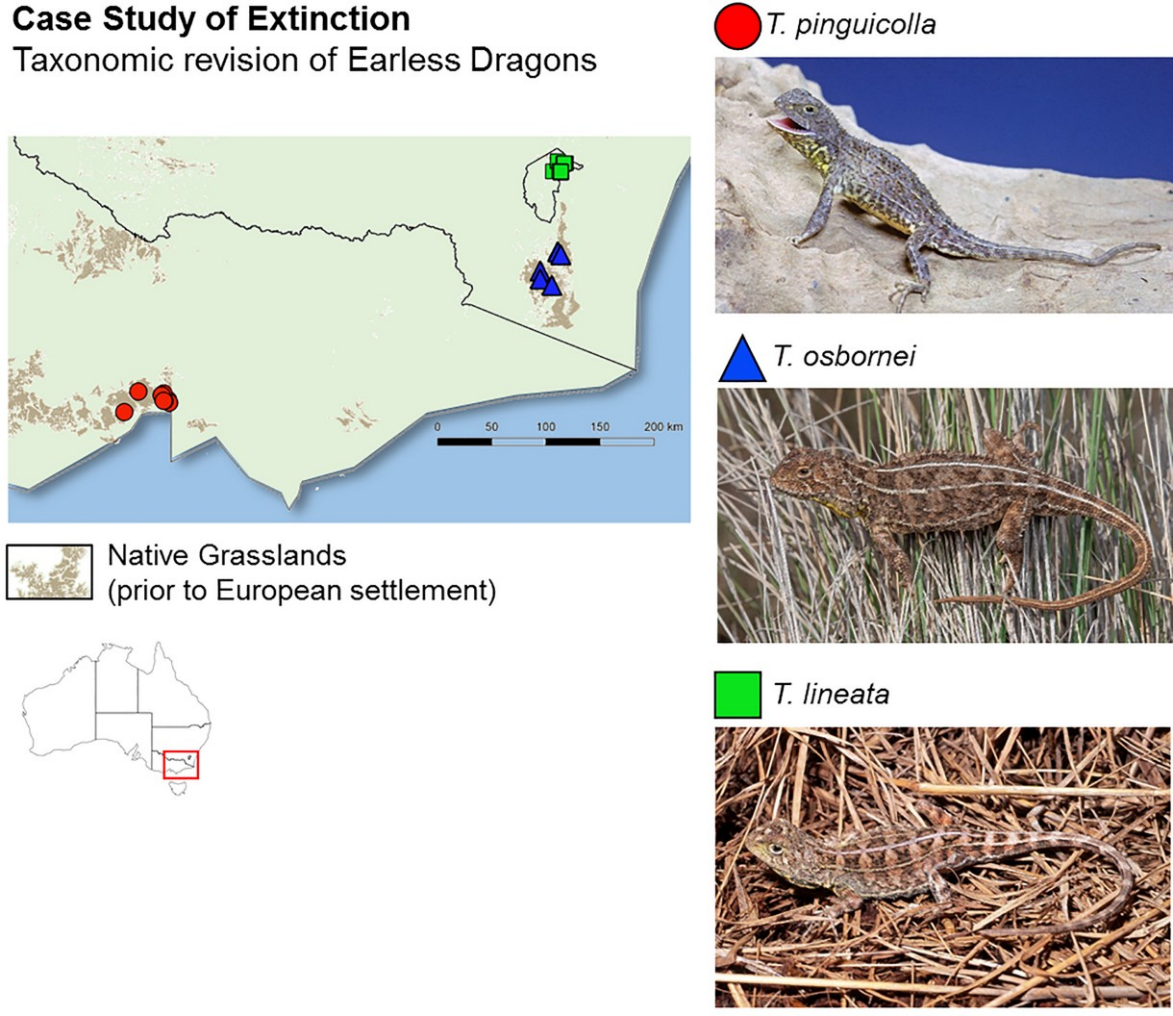
Earless Dragons—A case study of taxonomic research in groups containing undescribed species with a high probability of being threatened. The Grassland Earless Dragon (*Tympanocryptis* spp.) species complex occurred in the temperate native grasslands of south-eastern Australia (main map), grouped within a single species (*Tympanocryptis pinguicolla*), which was listed as Endangered on the IUCN Red List. However, available genetic and genomic data provided strong evidence that a full taxonomic revision was warranted [10,22]. As a result, *T*. *pinguicolla* is now thought to have been restricted to the Melbourne region and may represent the first extinction of a reptile on mainland Australia. Two new species, *Tympanocryptis osbornei* and *Tympanocryptis lineata*, were described and have a high probability of a threatened status. Full details of this case study and an example ROI assessment are provided in the supplementary materials (S1 Text, S3 Fig, S3 Table). Image credits: *T*. *pinguicolla* (F. and C. Collet); *T*. *osbornei* and *T*. *lineata* (S. Wilson). Map layer: Bioregional Assessment Source Dataset (https://data.gov.au/data/dataset/0cb242e2-daed-4507-a42e-73892c0941a1). Vegetation layer: pre-1750 tussock grasslands Department of Environment and Energy. 2018. National Vegetation Information System (NVIS) Version 5.1—AUSTRALIA (https://www.environment.gov.au/land/native-vegetation/national-vegetation-information-system/data-products).

A continental-scale approach such as this can provide crucial information for targeting regions of high need for taxonomic research. However, this ROI assessment approach is easily scaled down to focus on a single species complex. We provide a case-study in a species complex of earless dragons (*Tympanocryptis* spp.) to demonstrate how this approach can also be applied at a smaller scale (S1 Text). In south-eastern Australia, earless dragons in the critically endangered temperate grasslands provide a powerful example of how candidate species can sit hidden and unrecognized to the detriment of conservation outcomes. A recent taxonomic revision [10,22] determined grassland earless dragons comprise a number of candidate species with a high probability of threatened status (Fig 3). One of these newly revised species may represent the first extinction of a reptile on mainland Australia—a probable extinction that occurred prior to taxonomic recognition [10]. Such sobering examples of species being described after they have gone extinct are surprisingly common and not restricted to particular faunal groups or geographic regions [23–26]. A quantitative approach, such as the one we outline here that identifies and prioritizes species for rigorous taxonomic research, may help prevent such occurrences in the future.

**Fig 4:**
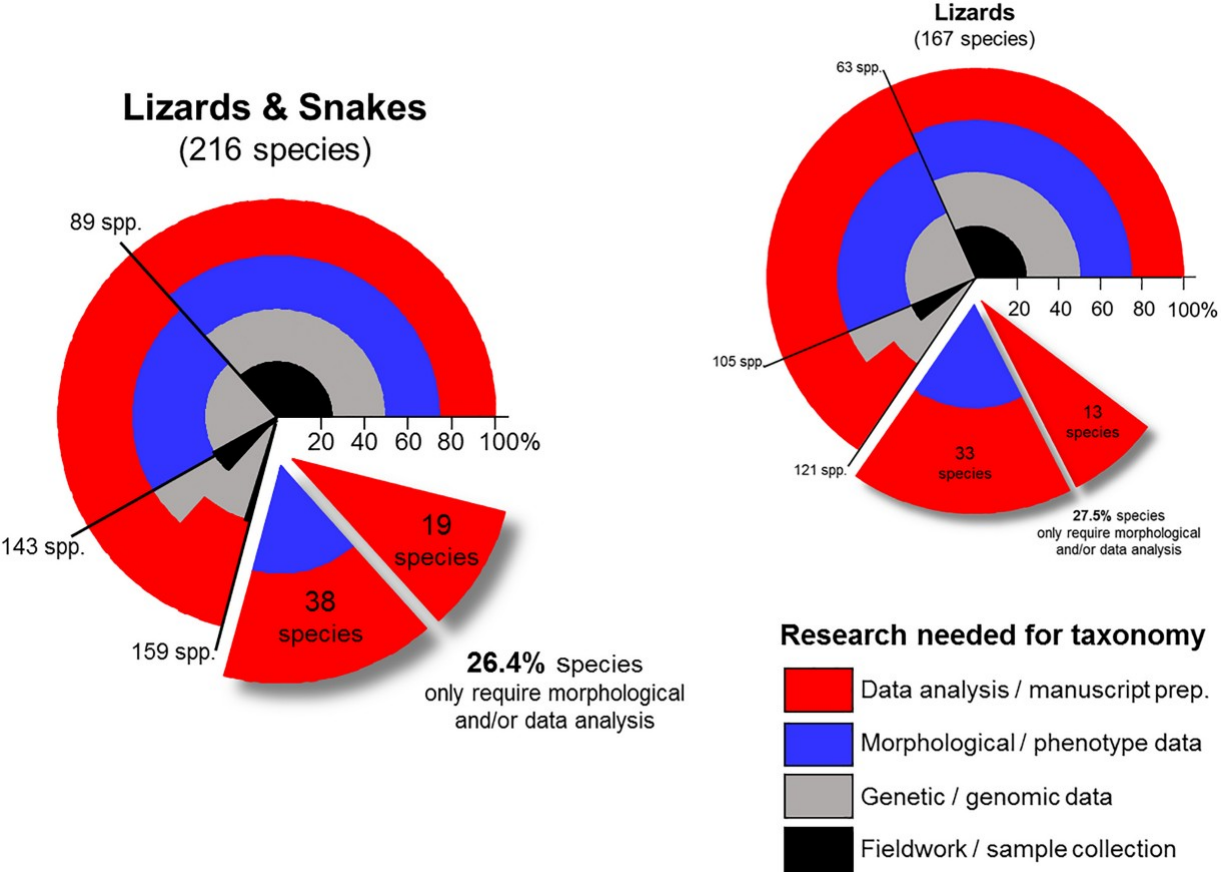
Research needed for taxonomy of Australian lizards and snakes. Polar graphs represent the proportion of the 4 research categories needed to complete taxonomic revision of a given species. All species assessed as probably or definitely needing taxonomic revision that would lead to increased diversity are included. Cumulative species count is provided around the exterior of the polar graphs. Two sections of the graphs are displaced to highlight the proportion of species for which the genetics/genomic research has already been completed. Of the 49 snake species containing undescribed species, 9 already have data available, only requiring analysis and manuscript preparation. Assessment data underlying figure available in S1 Data.

Globally, there is a significant backlog of species awaiting description across most organismal groups, largely due to the lack of resources to undertake taxonomic projects [27]. Recent large scale genomic and bar-coding projects are rapidly uncovering new species, with a $180 million global effort to identify more than 10 million new species [28]. These genetic initiatives provide a powerful approach to understanding global diversity, yet this is only the first step in the formal recognition of species. In the Australian squamates alone, there is a backlog of 26.4% of candidate species for which field samples and genetics/genomics data have been collected, but essential elements of taxonomic research are awaiting completion (Fig 4). The process of describing and naming species—taxonomy—requires multiple steps beyond genetic delineation, including comprehensive morphological/phenotypic/diagnostic assessment combined with a high level of familiarity and scholarship of the group in question [29]. To make a difference, resources need to be invested in taxonomy, including research funding and increased provision of viable career-paths [30,31].

Without taxonomic research, the conservation assessment and management of unrecognized species will not proceed [32]. There are untold numbers of species globally that are undocumented and are already under threat of extinction, possibly going extinct before we have the opportunity to save them. Our study demonstrates that large-scale approaches that unify taxonomic expertise and incorporate a return-on-investment methodology provide a powerful argument for targeting effort and resources. Such return-on-investment methods are well established in conservation biology for prioritization of conservation efforts and provide a promising new approach for targeting rigorous taxonomic research across many faunal groups.

## Methods

### Structured expert elicitation

We gathered a panel of experts on Australian squamate taxonomy and systematics. This panel worked on developing an easy to implement prioritization method to identify species of greatest need for rigorous taxonomic research in terms of conservation outcomes. The core of our expert panel was the Taxonomic Advisory Committee of the professional association, the Australian Society of Herpetologists, with additional panel members identified as being the taxonomic experts on specific squamate groups. Ten members of this expert panel had also participated in the 2017 IUCN Red List assessment workshops that assessed the conservation status of Australian squamates and were well versed in the process and guidelines for determining the various threatened categories.

During these 2017 IUCN workshops, researchers provided expert assessments on currently recognized species. The IUCN assessment process is focused at the species level, although some sea turtles have some subpopulation-level assessments—mammals and birds have a higher incidence of subspecific assessments. Of 983 IUCN Red List assessments from Australia, Christmas Island, and Norfolk Island, 60 species (6.3% of assessments) are recorded as requiring further taxonomic research (available at https://www.iucnredlist.org/, Version 2019–2), spanning those that are Data Deficient (6 species), Endangered (2 species), Near Threatened (1 species), Vulnerable (2 species), and Least Concern (49 species). This level of taxonomic research needed in Australian squamates is somewhat lower than that recorded for squamates globally, with 900 listed as needing taxonomic work (12.4% of 7,259 squamates). It is unclear why there is a lower level of taxonomic research need recorded in Australia. Our experts anecdotally believed this was an underestimate of taxonomic uncertainties, either at the 6.3% of Australian squamates listed as currently requiring taxonomic research or the global average of 12.4%.

Consequently, we developed a set of questions that aimed to determine whether taxonomic revision was likely to be required for a given species and if these revisions were considered likely to result in an increase or decrease in species diversity. Some species were deemed to be extremely complex, characterized as being a taxonomic “mess” without enough current information to reasonably predict an outcome. These species were put into a third category: “species complex.” Only those species categorized as leading to an increase in diversity were used in subsequent analyses.

We used 2 approaches to limit overinflation in our estimation of taxonomic uncertainty. Firstly, experts were asked to provide the level of confidence that taxonomic revision is needed, including: no revision, probably not, highly probable, and definitely. We then only used the last 2 categories in our analyses. Secondly, we used a binary system for categorizing whether taxonomic revision would lead to an increase, decrease, or a “species complex,” where 1 = yes and 0 = no. This is opposed to trying to estimate exactly how many species are contained within the current taxa. Our binary approach means that even if there are a small percentage of the “highly probable” and “definitely” categories that do not end up leading to an increase in diversity, it is only an overestimate of “1” for each case. This approach means that our assessment is a conservative one, as there are numerous species that contain multiple undescribed species, but with our binary methodology, they are counted as “1.”

Experts then provided information on what research was needed to complete the taxonomic revision of the species, separated into separate categorical items: (i) fieldwork and additional sampling; (ii) genetics and/or genomics; (iii) morphological and/or other phenotypic data; and (iv) analysis and manuscript preparation. Again, we used a binary coding system for each of these categories, where 1 = yes and 0 = no.

In conjunction with these taxonomic questions, we also asked for information regarding the predicted conservation concern of any new species that would results from the taxonomic revisions assessed above. To assess this, we asked for information on 3 factors: distribution size; known presence of threatening processes; and current conservation status of “parent” species. Firstly, we asked whether there would be any highly localized species, defined as those species with an extent of occurrence smaller than 20,000 km^2^. This is the IUCN Red List threshold for being eligible to be considered Vulnerable, putting a species at greater risk from threatening processes. We used a binary coding system for each of these categories, where 1 = yes and 0 = no. Secondly, asked experts to predict whether any of the candidate species would be the subject of species-level threatening processes (as defined by the IUCN Red List), and thirdly, if there would be a high probability of a candidate species being assessed as Vulnerable (VU), Endangered (EN), or Critically Endangered (CR). Species were scored as having a high probability of containing undescribed threatened species if they were already VU, EN, or CR, or if there was a closely related and very similar species to the undescribed taxa that was listed as VU, EN, or CR. For each of these categories, threatening processes and high probability of being threatened, we used a binary coding system, where 1 = yes and 0 = no.

Experts were asked to provide data only on those species for which they were able to give a meaningful, expert assessment. Free text cells were also provided for each section—taxonomy, research components, and predicted conservation concern—allowing further explanation of decisions. If experts were unable to provide a prediction of conservation concern, a conservative approach was taken and this section was scored as “0” (no concern).

To avoid influence of other expert opinions, spreadsheets without any assessment data were sent to each research group. The spreadsheets provided a list of all current species (rows) and columns for each of the taxonomic, research, and conservation criteria to be assessed. When spreadsheets were returned with assessment data, they were compiled by JM and JS. Any species for which there were multiple assessments sent in from different research groups were checked to see if they agreed. There was differing assessment in the need for taxonomic revision for 27 species, which fell into 3 categories: (1) morphological variation suggests potential candidate species, but unpublished genetic/genomic data suggest within species morphological variation (21 species); (2) morphological variation suggests potential geographically isolated, short-range endemic candidate species but no genetic/genomic data available for that location (5 species); and (3) genetic/genomic data provide strong evidence of short-range endemic candidate species; however, an assessor was unaware that a recent taxonomic publication has already described this species (1 species). Each of these cases was followed up with the researchers to gain more detail on the information they were using to provide this assessment and the confidence in the level of divergence, particularly for the second category, which was based on knowledge of morphological variation. Using a conservative approach, we coded those species in category (1) and (3) as not requiring taxonomic revision, and those in category (2) as having a high probability of taxonomic revision needed.

Expert assessment was provided for 870 species of the 1,034 currently recognized Australian squamates (assessment provided in S1 Data). Of the assessments that were categorized as highly probable or definitely needing taxonomic revision, 273 were based on some form of genetic/genomic evidence, while 8 were based on morphological variation in geographically defined parts of a range. Summary data of assessments, used in further analysis, is provided in S1 Table. We provide an example species assessment on available data in the supplementary materials (S1 Text, S3 Fig, S3 Table).

### Return-on-investment analyses

Return-on-investment (ROI) is a cost-effectiveness tool that has been used in conservation as a prioritization approach evaluating benefits against costs [17,33–35]. In contrast to the benefit–cost analysis (BCA), ROI typically examines a larger scale (e.g., a region or country) and does not does not require conversion of benefits into monetary equivalents [35]. It provides a more objective, transparent, and data-driven approach to evaluation, without the need to monetize analytical steps. In its simplest form, ROI analysis is estimated by dividing the conservation benefit of a particular action by the cost of taking that action [17]. As yet, ROI analysis has not been used in evaluating the role of taxonomic research in conservation. In our study, we applied an approach using a categorical score for the research steps needed to finalize taxonomic work (*r*1 → *r*4) on a given species as the relative “costs.” We then applied a categorical score to the predicted conservation features of the taxonomic work (*c*1 → *c*4) as the relative “benefits” for that species. We also incorporated a measure of certainty as to whether taxonomic revision is required for a given species, from no revision needed (*tax =* 0), probably not (*tax =* 0.2), highly probable (*tax =* 0.8), and definitely (*tax* = 1.0). Initially, we excluded all species with a *tax* of 0. We also only included those species that had been identified as requiring taxonomic revision that would lead to an increase in species numbers. All resulting species were included in the ROI analysis (*N =* 216). ROI for each species was calculated as follows:

$$ROI=\frac{\left(\sum c1\to c4\right)\times \mathrm{tax}}{\sum r1\to r4}$$

where *c*1 → *c*4 denote predicted conservation features of species described in taxonomic research: *c*1, taxonomic description allows conservation assessment; *c*2, highly localized candidate species (defined as those species with an extent of occurrence smaller than 20,000 km^2^); *c*3, candidate species would be the subject of species-level threatening processes (as defined by the IUCN Red List); and *c*4, high probability of a candidate species being assessed as Vulnerable, Endangered, or Critically Endangered (determined if parent species or close relative with similar traits were already listed as VU, EN, or CR).

Research steps required to complete the taxonomic work (*r*1 → *r*4) were defined as:

*r*1, fieldwork and additional sampling; *r*2, genetics and/or genomics; *r*3, morphological and/or other phenotypic data; and *r*4, analysis and manuscript preparation.

S1 Fig illustrates the decision framework in this ROI analysis, detailing the categorical score attributed to each factor.

### Geographic patterns of taxonomic assessment, predicted conservation concern, and ROI

During two 5-day IUCN workshops that were held in Australia to assess the extinction risk of Australian terrestrial squamates against IUCN criteria (Perth, February 2017; Melbourne, June 2017), distribution maps were compiled for 948 species. Distributional data used for mapping were collated by Tingley and colleagues [19] and are publically available from the IUCN website (https://www.iucnredlist.org/). The result of this process was a refined geographic range polygon for each species, converted to a shapefile and clipped to the Australian coastline (data available from https://www.iucnredlist.org/). These shapefiles were used to generate geographic distribution maps for the taxonomic, predicted conservation, and ROI data.

Species geographic range maps were overlaid on a 25 km × 25 km grid to estimate spatial patterns of species richness in each grid cell for: (i) squamate species for which taxonomic revision would lead to an increase in diversity; (ii) species that are predicted to contain undescribed highly localized species; (iii) species that are predicted to contain undescribed threatened species; and (iv) the estimate of ROI for species. We mapped species richness of features (i) and (ii) in each grid cell. For features (iii) and (iv), we present species richness and means of those scores for each 25-km grid cell. For example, if 6 species were present in a grid cell, of which 4 had an ROI of 1, one of 3 and one of 4, the species would equal the total number of species in the cell [6], whereas the weighted mean would be 1.83 (11/6). The latter approach accounted for overall species richness in a cell, allowing identification of regions with high ROI scores but low species diversity. We repeated all the above analyses at 1 km resolution for Lord Howe Island (group) and Norfolk Island (group). This finer spatial resolution was used to better visualize geographic patterns, given the relatively small spatial extent of the islands. The weighted mean of ROI was found to be influenced by a small number of high-scoring map cells (see S2A and S2B Fig). Consequently, weighted-mean of ROI in each cell was square root transformed to negate the influence of the low number of high-scoring cells (see S2C Fig).

## Supporting information

S1 Data Excel file of taxonomic assessment of Australian lizards and snakes. (XLSX)

S1 Fig Decision framework in the return on investment analysis (ROI). Categorical scores attributed to each factor are provided.
(DOCX)

S2 Fig Geographical distribution of mean score of return on investment analysis (ROI). (a) map of raw mean ROI score, with insets (not to same scale) showing Norfolk Island group (A) and Lord Howe Island group (B); (b) the frequency of raw mean ROI scores in mapping cells; and (c) the frequency of square root transformed mean ROI scores in mapping cells. Graph (b) shows a non-normal distribution with a small number of high ROI scores, where square root transformation of cell means improves the distribution (c). Map layer: Bioregional Assessment Source Dataset (https://data.gov.au/data/dataset/0cb242e2-daed-4507-a42e-73892c0941a1).
(DOCX)

S3 Fig Geographic distribution of species described from the *Tympanocryptis lineata* species group. A more detailed description and account of the south-eastern Grassland Earless Dragons is provided in the main paper (Fig 3). Map layer: Bioregional Assessment Source Dataset (https://data.gov.au/data/dataset/0cb242e2-daed-4507-a42e-73892c0941a1). Vegetation layer: pre-1750 tussock grasslands Department of Environment and Energy. 2018. National Vegetation Information System (NVIS) Version 5.1—AUSTRALIA (https://www.environment.gov.au/land/native-vegetation/national-vegetation-information-system/data-products).
(DOCX)

S1 Table Summary of taxonomic assessment of Australian squamates. All values are numbers of species. Data displayed are for species identified as “high probability” or “definitely” taxonomic work required. Predicted taxonomic outcomes are categorized as leading to an “Increase” or “Decrease” in species number or if it is a “Species Complex” for which the species boundaries and diversity if very complex and too difficult to currently predict. Families with number of assessed species that are <75% of total are highlighted—indicating panel was unable to provide expert assessment on a high proportion of species. Families with ≥30% of assessed species would lead to an increase in diversity are highlighted as groups requiring high levels of taxonomic revision.
(DOCX)

S2 Table Summary of taxonomic assessment of Australian freshwater turtles. All values are numbers of species. Data displayed are in the same format as that detailed in S1 Table.
(DOCX)

S3 Table Example of ROI assessment based on available data prior to taxonomic revision. (DOCX)

S1 Text Case study of an ROI assessment process. (DOCX)
