# Supplementary material for: A return-on-investment approach for prioritization of rigorous taxonomic research needed to inform responses to the biodiversity crisis
Source: PLoS Biol. 2021 Jun 1;19(6):e3001210. doi: 10.1371/journal.pbio.3001210 (PMC8168848; doi:10.1371/journal.pbio.3001210)
Supplement: S1 Fig — Categorical scores attributed to each factor are provided. (DOCX) [file pbio.3001210.s002.docx]

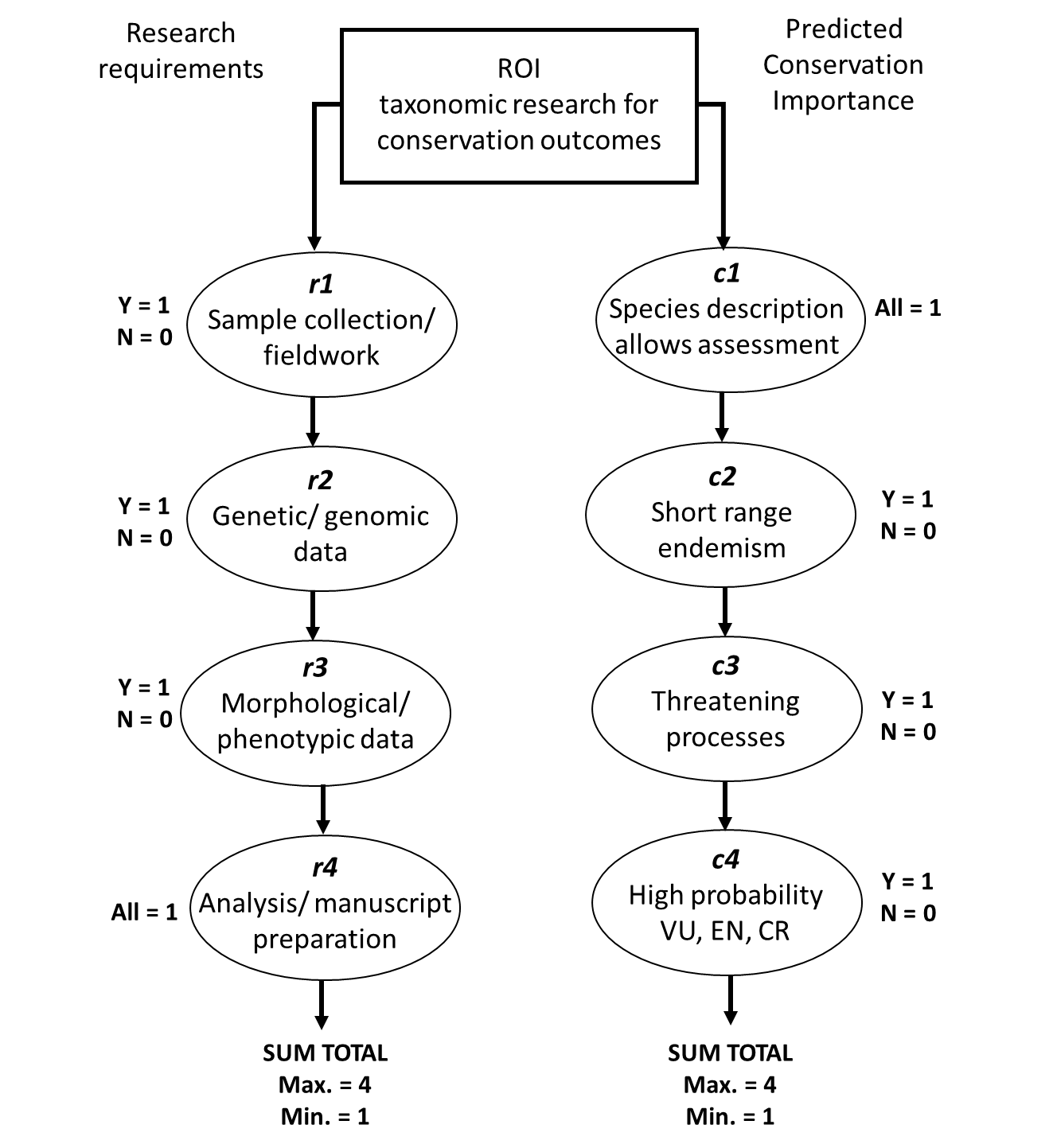


S1 Fig. Decision framework in the return on investment analysis (ROI). Categorical scores attributed to each factor are provided.
