# Supplementary material for: A return-on-investment approach for prioritization of rigorous taxonomic research needed to inform responses to the biodiversity crisis
Source: PLoS Biol. 2021 Jun 1;19(6):e3001210. doi: 10.1371/journal.pbio.3001210 (PMC8168848; doi:10.1371/journal.pbio.3001210)
Supplement: S2 Fig — (a) map of raw mean ROI score, with insets (not to same scale) showing Norfolk Island group (A) and Lord Howe Island group (B); (b) the frequency of raw mean ROI scores in mapping cells; and (c) the frequency of square root transformed mean ROI scores in mapping cells. Graph (b) shows a non-normal distribution with a small number of high ROI scores, where square root transformation of cell means improves the distribution (c). Map layer: Bioregional Assessment Source Dataset (https://data.gov.au/data/dataset/0cb242e2-daed-4507-a42e-73892c0941a1). (DOCX) [file pbio.3001210.s003.docx]

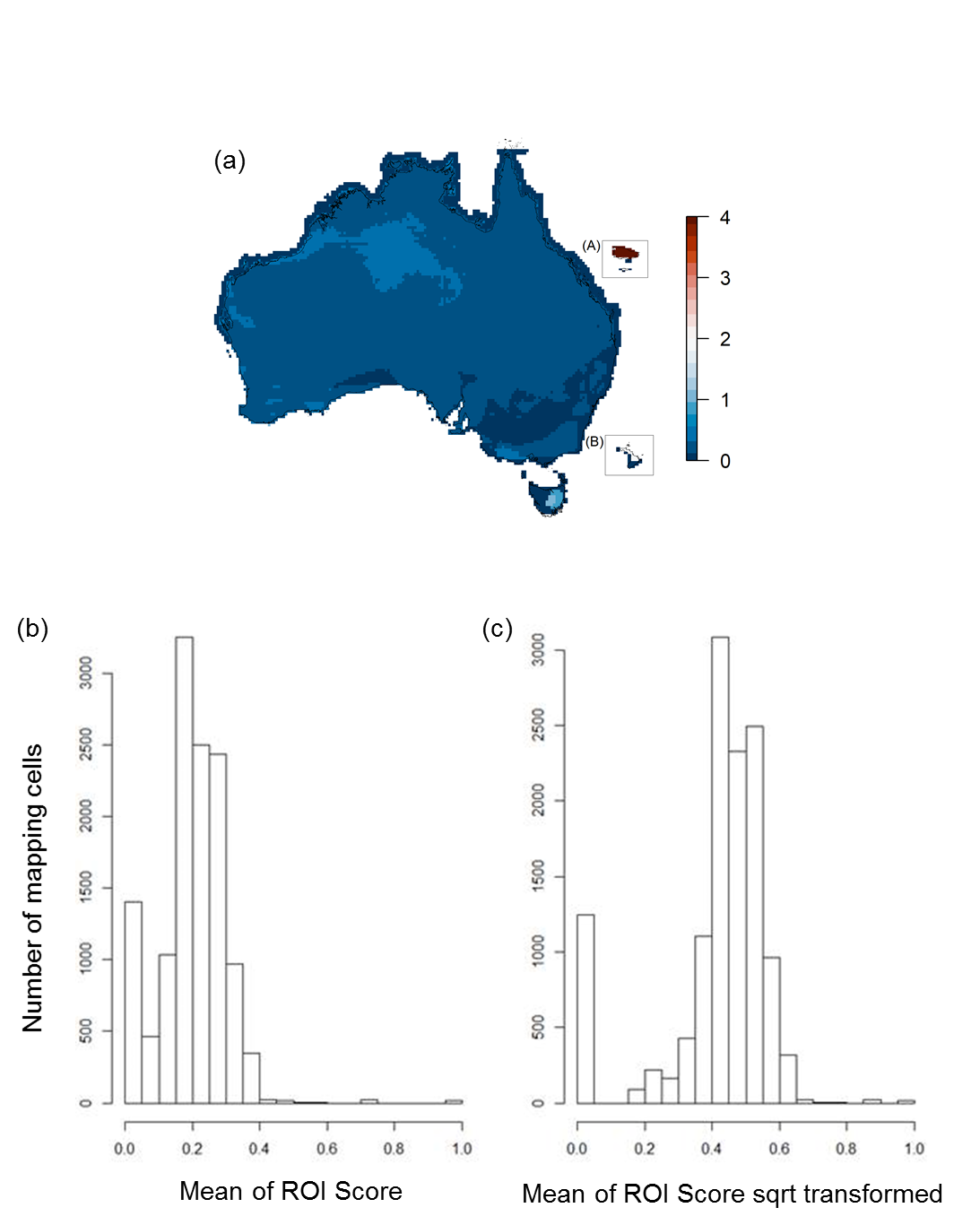


**S2 Fig.** **Geographical distribution of mean score of return on investment analysis (ROI):** (a) map of raw mean ROI score, with insets (not to same scale) showing Norfolk Island group (A) and Lord Howe Island group (B); (b) the frequency of raw mean ROI scores in mapping cells; and (c) the frequency of square root transformed mean ROI scores in mapping cells. Graph (b) shows a non-normal distribution with a small number of high ROI scores, where square root transformation of cell means improves the distribution (c). Map layer: Bioregional Assessment Source Dataset (https://data.gov.au/data/dataset/0cb242e2-daed-4507-a42e-73892c0941a1).
