# Supplementary material for: A return-on-investment approach for prioritization of rigorous taxonomic research needed to inform responses to the biodiversity crisis
Source: PLoS Biol. 2021 Jun 1;19(6):e3001210. doi: 10.1371/journal.pbio.3001210 (PMC8168848; doi:10.1371/journal.pbio.3001210)
Supplement: S3 Fig — A more detailed description and account of the south-eastern Grassland Earless Dragons is provided in the main paper (Fig 3). Map layer: Bioregional Assessment Source Dataset (https://data.gov.au/data/dataset/0cb242e2-daed-4507-a42e-73892c0941a1). Vegetation layer: pre-1750 tussock grasslands Department of Environment and Energy. 2018. National Vegetation Information System (NVIS) Version 5.1—AUSTRALIA (https://www.environment.gov.au/land/native-vegetation/national-vegetation-information-system/data-products). (DOCX) [file pbio.3001210.s004.docx]

**
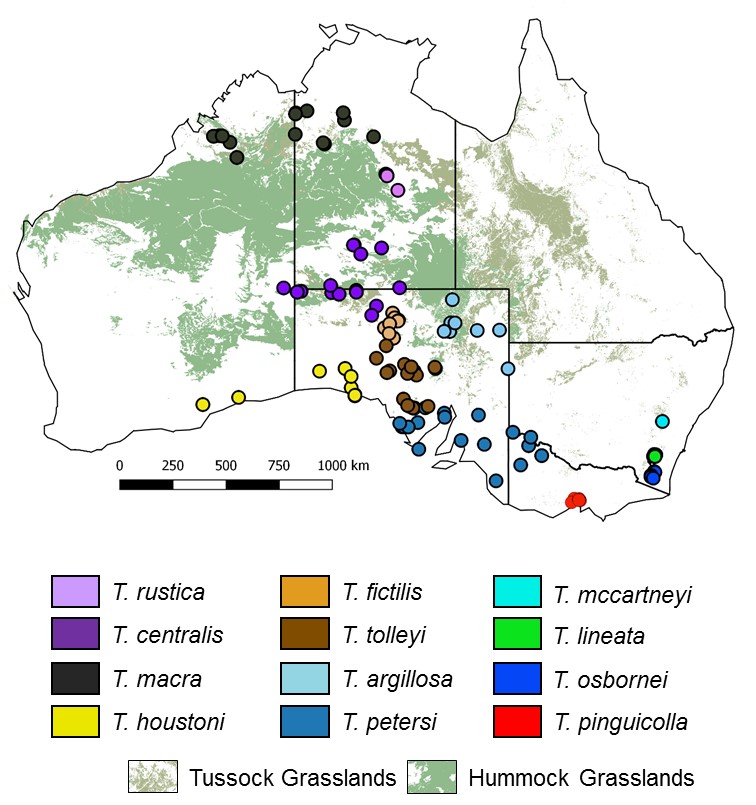
**

**Fig S3. Geographic distribution of species described from the *Tympanocryptis lineata* species group.** A more detailed description and account of the south-eastern Grassland Earless Dragons is provided in the main paper (Fig 3). Map layer: Bioregional Assessment Source Dataset (<https://data.gov.au/data/dataset/0cb242e2-daed-4507-a42e-73892c0941a1>). Vegetation layer: pre-1750 tussock grasslands Department of Environment and Energy. 2018. National Vegetation Information System (NVIS) Version 5.1 - AUSTRALIA (https://www.environment.gov.au/land/native-vegetation/national-vegetation-information-system/data-products).
