# Supplementary material for: A return-on-investment approach for prioritization of rigorous taxonomic research needed to inform responses to the biodiversity crisis
Source: PLoS Biol. 2021 Jun 1;19(6):e3001210. doi: 10.1371/journal.pbio.3001210 (PMC8168848; doi:10.1371/journal.pbio.3001210)
Supplement: S1 Table — All values are numbers of species. Data displayed are for species identified as “high probability” or “definitely” taxonomic work required. Predicted taxonomic outcomes are categorized as leading to an “Increase” or “Decrease” in species number or if it is a “Species Complex” for which the species boundaries and diversity if very complex and too difficult to currently predict. Families with number of assessed species that are <75% of total are highlighted—indicating panel was unable to provide expert assessment on a high proportion of species. Families with ≥30% of assessed species would lead to an increase in diversity are highlighted as groups requiring high levels of taxonomic revision. (DOCX) [file pbio.3001210.s005.docx]

**S1 Table**. Summary of taxonomic assessment of Australian squamates. All values are numbers of species. Data displayed are for species identified as “high probability” or “definitely” taxonomic work required. Predicted taxonomic outcomes are categorized as leading to an “Increase” or “Decrease” in species number or if it is a “Species Complex” for which the species boundaries and diversity if very complex and too difficult to currently predict. Families with number of assessed species that are <75% of total are highlighted – indicating panel was unable to provide expert assessment on a high proportion of species. Families with ≥30% of assessed species would lead to an increase in diversity are highlighted as groups requiring high levels of taxonomic revision.

|  | Total  Species | Number Assessed | Increase | Decrease | Species Complex | Fieldwork/Sampling | Genetics/Genomics | Morphology/Phenotypic | Short Range Endemics | Threatening Processes | Endangered  High Probability |
| --- | --- | --- | --- | --- | --- | --- | --- | --- | --- | --- | --- |
| **Lizards** |  |  |  |  |  |  |  |  |  |  |  |
| Agamidae | 97 | 97 | 17 | 0 | 1 | 5 | 7 | 14 | 5 | 4 | 3 |
| Carphodactylidae | 31 | 31 | 5 | 1 | 0 | 4 | 5 | 5 | 0 | 1 | 0 |
| Diplodactylidae | 95 | 93 | **37** | 1 | 5 | 18 | 35 | 28 | 7 | 1 | 3 |
| Gekkonidae | 63 | 57 | 6 | 0 | 0 | 1 | 5 | 6 | 1 | 2 | 0 |
| Pygopodidae | 44 | 42 | 6 | 1 | 0 | 0 | 7 | 7 | 1 | 0 | 1 |
| Scincidae | 458 | 378 | 86 | 12 | 28 | 66 | 87 | 109 | 32 | 11 | 5 |
| Varanidae | 30 | 30 | **10** | 4 | 1 | 5 | 11 | 1 | 4 | 6 | 0 |
|  |  |  |  |  |  |  |  |  |  |  |  |
| **Snakes** |  |  |  |  |  |  |  |  |  |  |  |
| Elapidae | 138 | **84** | 23 | 3 | 7 | 12 | 20 | 16 | 0 | 0 | 0 |
| Pythonidae | 15 | 15 | **5** | 0 | 1 | 2 | 5 | 2 | 0 | 1 | 0 |
| Typhlopidae | 47 | **33** | **14** | 1 | 0 | 10 | 8 | 14 | 2 | 0 | 0 |
| Other snakes | 16 | 10 | **7** | 0 | 0 | 7 | 8 | 7 | 0 | 0 | 0 |
|  |  |  |  |  |  |  |  |  |  |  |  |
| **Total** | 1034 | 870 | 216 | 23 | 43 | 130 | 98 | 209 | 52 | 26 | 12 |
|  |  |  |  |  |  |  |  |  |  |  |  |
