# Supplementary material for: A return-on-investment approach for prioritization of rigorous taxonomic research needed to inform responses to the biodiversity crisis
Source: PLoS Biol. 2021 Jun 1;19(6):e3001210. doi: 10.1371/journal.pbio.3001210 (PMC8168848; doi:10.1371/journal.pbio.3001210)
Supplement: S2 Table — All values are numbers of species. Data displayed are in the same format as that detailed in S1 Table. (DOCX) [file pbio.3001210.s006.docx]

**S2 Table**. Summary of taxonomic assessment of Australian freshwater turtles. All values are numbers of species. Data displayed are in the same format as that detailed in Table S1.

|  | Total  Species | Number Assessed | Increase | Decrease | Species Complex | Fieldwork/Sampling | Genetics/Genomics | Morphology/Phenotypic | Short Range Endemics | Threatening Processes | Endangered  High Probability |
| --- | --- | --- | --- | --- | --- | --- | --- | --- | --- | --- | --- |
| **Turtles** |  |  |  |  |  |  |  |  |  |  |  |
| Chelidae | 23 | 23 | 6 | 0 | 0 | 0 | 1 | 1 | 0 | 1 | 0 |
|  |  |  |  |  |  |  |  |  |  |  |  |
