# Supplementary material for: A return-on-investment approach for prioritization of rigorous taxonomic research needed to inform responses to the biodiversity crisis
Source: PLoS Biol. 2021 Jun 1;19(6):e3001210. doi: 10.1371/journal.pbio.3001210 (PMC8168848; doi:10.1371/journal.pbio.3001210)
Supplement: S3 Table — (DOCX) [file pbio.3001210.s007.docx]

S3 Table. Example of ROI assessment based on available data prior to taxonomic revision.

|  | *T. lineata* | *T. pinguicolla* | *T. houstoni* | *T. centralis* |
| --- | --- | --- | --- | --- |
| **Taxonomic revision needed?^a^ (*tax*)** | 1.0 | 1.0 | 0 | 0.8 |
|  |  |  |  |  |
| **Research Needed** |  |  |  |  |
| Samples (*r1*) | 0 | 0 | 0 | 0 |
| Genetics/Genomics (*r2*) | 0 | 0 | 0 | 0 |
| Morphology (*r3*) | 1 | 1 | 1 | 1 |
| Analysis/Manuscript Prep (*r4*) | 1 | 1 | 1 | 1 |
| **Sub-total** | **2** | **2** | **2** | **2** |
|  |  |  |  |  |
| **Conservation** |  |  |  |  |
| Description allows assessment (*c1*) | 1 | 1 | 1 | 1 |
| Short-range endemic (*c2*) | 0 | 1 | 0 | 0 |
| Likely threatening processes (*c3*) | 1 | 1 | 0 | 0 |
| High probability of threatened (*c4*) | 0 | 1 | 0 | 0 |
| **Sub-total** | 2 | 4 | 1 | 1 |
|  |  |  |  |  |
| ROI calculation | (2*1)/2 | (4*1)/2 | (2*0)/1 | (1*0.8)/2 |
| **ROI score** | **1.0** | **2.0** | 0 | 0.4 |

^a^ Taxonomic revision multiplier: 1.0 = definitely, 0.8 = highly probably, 0.2 = probably not, 0 = no
