# Supplementary material for: A return-on-investment approach for prioritization of rigorous taxonomic research needed to inform responses to the biodiversity crisis
Source: PLoS Biol. 2021 Jun 1;19(6):e3001210. doi: 10.1371/journal.pbio.3001210 (PMC8168848; doi:10.1371/journal.pbio.3001210)
Supplement: S1 Text — (DOCX) [file pbio.3001210.s008.docx]

S1 Text. Case Study of an ROI Assessment process

Here we provided a detailed example of the assessment process that was undertaken on all Australian squamates. Here we provide: (1) details of information available prior to taxonomic research on which our prioritization method could have been used; (2) how the ROI method would have been implemented using this information (Table S3); and (3) how subsequent taxonomic research impacted the future conservation management of this species group. This example is of a species group that has undergone taxonomic revision very recently (10, 22), with significant implications for conservation management. Note that this species group was not included in the overall estimates of outstanding taxonomic needs for Australian squamates, as taxonomic work is completed, but it provides a powerful example of the importance of such research.

**Background – Earless Dragons (*Tympanocryptis* spp.)**

The *Tympanocryptis lineata* species group, distributed in southeastern, central and north-western Australia (S3 Fig), was polyphyletic but united by the historical mishap that all were initially described as either a subspecies of *Tympanocryptis lineata* or have identified as local variations of the nominate subspecies (10). Prior to the recent taxonomic treatment (10, 22) there were three recognized species within this group (*T. lineata, T. houstoni* and *T. pinguicolla)* and a fourth (*T. centralis*) was informally recognized as a species in multiple publications^3^. Many of the southeastern locality records for this species group are from historic museum samples with no recent specimens or sightings over the last thirty to forty years. There have been significant declines in this species throughout the southeast and there are only a few areas to the east where the species is known to survive. For example, in South Australia around the Adelaide region it is regarded as Endangered (22). In north-western Victoria this species is now known from only five sites, where it is listed as Critically Endangered on the Advisory List of Threatened Vertebrate Fauna (22). The Grassland Earless Dragon (*T. pinguicolla*) was already listed as Endangered on the IUCN Red List prior to the recent taxonomic revision (10).

**Data Available Prior to Taxonomic Revision**

*Tympanocryptis lineata*: A combination of published and unpublished phylogeographic data spanning the complete range of this species complex was available for mtDNA and a nuclear gene (RAG1). These data provided strong evidence that there were deep species-level lineages across the range and that this group was paraphyletic. Field-based collections had revealed a number of populations that were strikingly different in color patterns and morphology (scale patterns). These same data suggested that there were deep genetic divergences within *T. centralis*. There was no evidence of species-level phylogenetic structure within *T. houstoni*. However, no overall assessment of morphology had been undertaken and it was unclear how field-observed morphological variation related to genetic data.

*Tympanocryptis pinguicolla*: Published genetic data suggested that there were deep, possibly species level divergences between historically isolated populations around Melbourne, Canberra and on the Monaro high plains (10, 22). Unpublished genomic (SNP) data provided strong support that the Canberra and Monaro populations were separate species. However, no overall assessment of morphology had been undertaken for any of the populations.

**Example of ROI assessment based on available data prior to taxonomic revision**

Using the data and information detailed above we undertake an example of the ROI process (S3 Table), in which we determine the research needed to complete a taxonomic revision of this species group and the predicted conservation importance of this taxonomic research. Finally, we apply the certainty multiplier, estimating our certainty of whether this work is needed. See method for full description of the scoring system.

Implementation of the ROI in the *Tympanocryptis lineata* species group identified two species of having a ROI of ≥1.0, indicating a high priority for rigorous taxonomic revision, with a high level of certainty of significant conservation implications.

**Outcome Following Taxonomic Revision**

Following taxonomic revision of this paraphyletic species complex, these four earless dragons have now been split up into 12 species (S3 Fig). If a prioritization assessment had been undertaken prior to this work *T. pinguicolla* would have been identified as a high priority species for immediate resourcing of taxonomic research. Based on the conservation concern about a candidate species within *T. lineata* this would also have been a priority taxon. Although the taxonomic identification and description of all candidate species is important and there are species of conservation concern for which additional fieldwork and genetic/genomic research is a priority, an ROI assessment method such as this allows the identification of those species of high conservation priority for which most data is already available.
